# A Deep Learning Approach for Microplastic Segmentation in Microscopic Images

**DOI:** 10.3390/toxics13121018

**Published:** 2025-11-25

**Authors:** Yuan Yao, Wending Xu, Haoxin Fan

**Affiliations:** 1 School of Computer Science and Engineering, Wuhan Institute of Technology, Wuhan 430205, China; 21120501@wit.edu.cn (Y.Y.); xuwending2025@163.com (W.X.); 2 School of Environmental Engineering, Wuhan Institute of Technology, Wuhan 430205, China

**Keywords:** microplastics, deep learning, image segmentation, environmental monitoring, lightweight neural networks

## Abstract

The ubiquitous presence of microplastics across environmental compartments presents a formidable ecotoxicological and risk assessment challenge, fundamentally complicated by the link between microplastic morphology and differential toxicological outcomes. Current analytical methods face a significant measurement bottleneck, hindering the precise, high-throughput characterization needed for robust mechanistic and exposure studies. To address this, we introduce MNv4-Conv-M-fpn, a novel deep learning model specifically engineered for multi-class microplastic segmentation and morphological characterization from microscopic images. This model is designed to provide the toxicologically-relevant granularity required for rigorous risk assessment, segmenting images into six classes: five distinct microplastic categories (fiber, fragment, sphere, foam, and film) and the background. By incorporating advanced architectural features—including transfer learning, a Feature Pyramid Network, and a Feature Fusion Module—our approach achieves high accuracy, computational efficiency, and near real-time inference speed. Comprehensive validation using a diverse dataset demonstrates that MNv4-Conv-M-fpn outperforms existing segmentation methods while maintaining low computational load. This makes the model well-suited for high-throughput deployment in environmental laboratories and resource-constrained monitoring efforts. This approach offers a valuable tool for environmental monitoring, enabling more precise and scalable analysis of microplastic pollution in various ecosystems.

## 1. Introduction

The global proliferation of plastics has resulted in the ubiquitous presence of plastic debris across all environmental compartments, from terrestrial systems to the deep sea [1]. A significant portion of this material fragments into microplastics (particles less than 5mm in size), which are now recognized as a critical pollutant with profound implications for ecotoxicology and environmental safety. This pollution is characterized by extreme persistence, as most plastics resist biodegradation and only break down slowly via mechanical and photochemical processes [2,3].

The escalating scale of microplastic pollution urgently necessitates robust measurement and analysis tools to investigate their occurrence, transport, and long-term toxicological effects on biota and ecosystems. Time series data from [4] reveal a significant increase in microplastic abundance in coastal waters. These particles are transported through complex, poorly understood pathways, from surface waters to the deep sea via processes like sediment gravity flows [5,6]. Risk assessments [7,8] confirm that many marine areas already face high ecological risks. These findings underscore the urgent need for robust measurement and analysis tools to investigate the sources, distribution, and long-term effects of microplastics on marine ecosystem structure and function.

The toxicological impacts of microplastic pollution are multifaceted, causing not only physical harm but also significant sublethal effects on biota across aquatic and terrestrial environments [9,10,11]. Furthermore, microplastics act as vectors for other environmental contaminants, thereby amplifying ecological risks within the marine environment [12]. A critical aspect of accurately assessing and managing these risks is the ability to accurately differentiate their physical and chemical characteristics. Specifically, morphological attributes (e.g., fibers, fragments, spheres) are crucial determinants of their environmental fate, transport dynamics, bioavailability, and ultimate toxicological effects [13]. Specifically, ref. [14] shows that the shape of these microplastics influences their mobility, buoyancy, interaction with marine organisms, and their broader impacts on health and biodiversity. Ref. [15] established that the shape of plastic debris is an essential factor for comprehensive categorization and pollution assessment.

To effectively characterize and quantify these diverse microplastic morphologies, researchers primarily utilize optical and electron microscopy. However, manual characterization under optical microscopy is inherently subjective, time-intensive, and prone to inconsistencies, severely limiting the throughput required for large-scale, comprehensive marine pollution monitoring [16]. To overcome these limitations and enhance the accuracy of environmental assessments, the adoption of deep learning and artificial intelligence (AI) for identifying plastic morphology in microscopy images is essential [17,18]. This automated approach is crucial for introducing new forms of measurement and analysis that can rapidly and precisely assess marine environmental conditions, thereby enabling better management and supporting policy decisions. For instance, the application of deep learning surrogate methods has been successfully demonstrated for predicting particle transport in coastal environments [19].

A key technique in this automation is image segmentation, which employs computer programs to divide microscopy images into distinct regions, labeling each pixel to identify and classify microplastics against the background. This allows researchers to rapidly process large volumes of environmental samples, providing critical data for management, such as size distributions and count statistics for each microplastic category. The work of [20] on seafloor debris detection using deep learning driven image restoration further validates the effectiveness of AI-enhanced image analysis in challenging environmental conditions.

Previous studies have explored the use of deep neural network models for microplastic image segmentation. These works are summarized in Table 1. The majority of these studies focus on binary segmentation, where images are categorized simply as microplastics versus background. For instance, ref. [21] employed a U-Net [22] to separate microplastics from background elements. Similarly, ref. [23] used MultiResU-Net [24], demonstrating improved accuracy. Other works have utilized architectures such as ResNet [25] combined with a feature pyramid network [17], various U-Net derivatives [26], and HRNet [27,28]. While some works have advanced toward multi-class segmentation, such as [29], which segmented beach and marine debris into five morphological categories, most remain focused on binary classification.

The fundamental limitation of most existing work is the focus on binary segmentation, which fails to capture the full diversity of microplastic types, each associated with distinct origins, transport mechanisms, and toxicological risks in the marine environment. This failure to provide morphological granularity hinders accurate exposure modeling and the development of targeted pollution control strategies. Our work addresses this critical gap in marine pollution measurement and analysis by tackling the technically more difficult problem of segmenting microplastics into five distinct morphological categories (plus the background). This high-performance multi-class segmentation approach allows for a significantly more nuanced and detailed assessment of contamination in environmental samples. Our accurate identification and segmentation of multiple microplastic types in microscope images highlight the potential of using this AI-driven approach as a new paradigm for versatile microplastic image segmentation. This level of morphological granularity is essential for tracking the distribution of microplastics and directly informing targeted pollution control and management strategies in the future.

## 2. Methodology

### 2.1. Deep Learning Model

**Figure 1:**
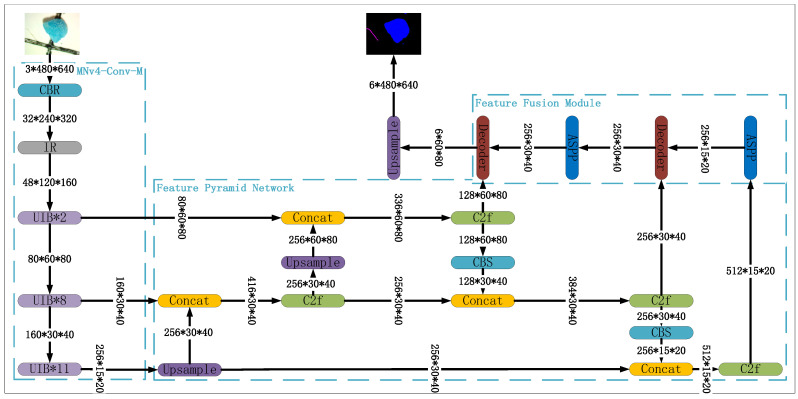
MNv4-Conv-M-fpn’s overall architecture.

The high-level architecture of the deep learning model proposed in this work is depicted in Figure 1. The model architecture is composed of a backbone network, a feature pyramid network (FPN), and a feature fusion module (FFM). In addition, we utilize a simple yet effective loss function to optimize our model. For simplicity, we will refer to our model as MNv4-Conv-M-fpn throughout the remainder of this paper. Our full implementation code is provided in https://github.com/xwd2019/MNv4-fpn/, accessed on 23 November 2025, with commit id 3b1976c.

#### 2.1.1. BackBone Network

In deep learning, a backbone network is a key part of the model architecture for tasks like semantic segmentation. It is typically a pre-trained convolutional neural network that extracts important features from input images. These features include edges, textures, and shapes, which help the model recognize and differentiate between various types of objects in the images, microplastics in this work. By using a backbone network, the model can efficiently perform complex image analysis with fewer resources.

The choice of a backbone network is crucial for balancing performance and computational cost. We employ MobileNetV4 [33] as the backbone for its lightweight and efficient architecture, which is specifically designed for resource-constrained environments. It leverages pre-training on large datasets and incorporates advanced architectural innovations, such as efficient channel attention mechanisms and optimized bottleneck layers, to achieve high segmentation accuracy with significantly reduced computational complexity. This efficiency, as demonstrated in Section 3, makes MobileNetV4 an ideal choice for high-throughput, environmental monitoring applications where accuracy and computational resources are critical.

The backbone network of our model is constructed using three submodules: CBR, IR, and UIB. These submodules are described as follows:

- CBR: Stands for Conv2d + Batch Normalization + ReLU, a widely used combination in deep learning models introduced in [34] that has proven highly effective in various image-related tasks.
- IR: Stands for Inverted Residual, introduced in [35]. The IR module applies the residual connection to high-dimensional feature maps. This inverted residual structure helps maintain the model’s performance even in relatively shallow network architectures.
- UIB: Stands for Universal Inverted Bottleneck, introduced in [33]. It builds on key components of MobileNetV4, specifically separable depthwise (DW) convolution and pointwise (PW) expansion and projection. UIB extends the Inverted Bottleneck (IB) block introduced in [35] and has become a standard building block for efficient neural network architectures.

#### 2.1.2. Feature Pyramid Network

Feature pyramid networks (FPNs), introduced in [36], are a series of powerful tools for handling objects of varying sizes, a critical challenge in microplastic image analysis. Unlike traditional methods that rely on a single scale of features, a feature pyramid network constructs a pyramid-like structure of several feature maps, each representing different levels of detail. By combining information from these multiple scales, an FPN effectively captures both fine-grained details necessary for locating small microplastics and broader context for understanding larger fragments. This multi-scale approach significantly enhances the ability to accurately detect and segment microplastics of diverse sizes within a single image, leading to improved overall performance.

A typical feature pyramid network is composed of the following components: Upsample, Concat, C2f, and CBS. These components can be described as follows:

- Upsample: upsamples the input tensor using the bilinear upsampling method;
- Concat: concatenates tensors along the channel dimension;
- C2f: Introduced in [37], this module uses two convolutional layers and a fusion layer. It takes feature maps from different spatial scales as input, then applies a series of convolution and concatenation operations to fuse these multi-scale feature maps, allowing the model to capture and integrate information from different spatial resolutions;
- CBS: Stands for Conv2d + Batch Normalization + SiLU. It is similar to the standard CBR module, but replaces the ReLU activation function with SiLU for potentially improved performance.

#### 2.1.3. Feature Fusion Module

The feature pyramid network employed in this work generates three feature maps, each capturing semantic information at different scales. Following this, the feature fusion module (FFM) plays a critical role in merging these feature maps into a cohesive representation that effectively balances fine details and broader semantic information. The FFM also includes mechanisms to prioritize the most relevant features, ensuring the final output is optimized for segmentation accuracy.

To further enhance the FFM’s performance, we integrate the Atrous Spatial Pyramid Pooling (ASPP) module and the decoder from DeepLabV3+ [38]. These components are essential for refining the features and boosting the model’s segmentation capabilities. These components can be described as follows:

- ASPP: Introduced in [39], atrous spatial pyramid pooling employs parallel atrous convolutions with varying dilation rates, enabling the model to capture features across multiple scales. This approach improves the model’s ability to handle objects of different sizes.
- Decoder: We directly incorporate the decoder from DeepLabV3+ [38], which combines the outputs from the ASPP module and FPN. This decoder upscales the feature maps, resulting in a richer and more detailed feature representation, ultimately contributing to more accurate segmentation.

#### 2.1.4. Transfer Learning

To boost the performance of our deep learning model for microplastic segmentation, we employed transfer learning as a crucial technique during training [40]. Specifically, we first pre-train our model on the Cityscapes dataset [41], which provided a rich set of features adaptable to the specific task of microplastic segmentation. After this pre-training, the model was fine-tuned using our microplastic image dataset.

The rationale behind this transfer learning approach is that the low-level features learned from the Cityscapes dataset, such as edge detection, texture recognition, and color identification, are likely to be useful for the microplastic task as well. As shown in Section 3.4, this transfer learning approach enhances the model’s performance by about 5%.

#### 2.1.5. Loss Function

A loss function measures the difference between a model’s predictions and the actual target values, guiding the optimization process during training. It quantifies how well or poorly the model is performing by calculating the error between predicted outputs and true values. For our case, where the main objective is to perform semantic segmentation of microplastics in images, the loss function evaluates how accurately the model identifies and classifies different microplastic types and how well it delineates their boundaries. If the model incorrectly classifies a fragment as a fiber or misses parts of a foam, the loss function reflects this discrepancy, prompting adjustments to improve the model’s accuracy and precision in detecting and segmenting microplastics.

Our model uses a combination of cross-entropy loss and Dice loss, each contributing uniquely to the training process.

The cross-entropy loss $L_{CE}$ is defined in Equation (1). In this equation, *N* is the total number of samples being evaluated. *M* is the number of classes of microplastics. $y_{ic}\in \left\{0,1\right\}$ is the label of sample *i* for class *c*, ${\hat{p}}_{ic}\in \left[0,1\right]$ is the predicted probability of sample *i* being in class *c* generated by the model. $w_{c}$ is the weight of each predicted class.

(1) $$\begin{array}{c} L_{CE}=-\frac{1}{N}\sum_{i=1}^{N}\sum_{c=1}^{M}w_{c}y_{ic}\log{\hat{p}}_{ic}. \end{array}$$

As described in [42], this loss function quantifies the dissimilarity between the predicted and actual categorical distributions, encouraging the model to assign higher probabilities to the correct class labels. By minimizing cross-entropy loss, the model improves its ability to accurately classify each pixel in the image into one of the predefined microplastic categories.

Dice Loss is computed using Equation (2). In Equation (2), ${\hat{y}}_{ic}\in \left\{0,1\right\}$ is the predicted label of sample *i* for class *c* by the model.

(2) $$\begin{array}{c} L_{Dice}=1-\frac{1}{N}\sum_{i}^{N}\sum_{c=1}^{M}\frac{2|y_{ic}⋂{\hat{y}}_{ic}|}{|y_{ic}\left|+\right|{\hat{y}}_{ic}|} \end{array}$$

As described in [43], this loss is particularly effective in tasks where the precise delineation of object boundaries is crucial. It is derived from the Dice coefficient, which is a measure of overlap between the predicted segmentation and the ground truth. By minimizing Dice loss, the model enhances its ability to produce accurate and contiguous segmentation masks, ensuring that the shapes and boundaries of microplastics are well-defined.

Combining cross-entropy loss and Dice loss, the loss function is defined in Equation (3).

(3) $$\begin{array}{c} L=\alpha L_{CE}+\left(1-\alpha \right)L_{Dice} \end{array}$$

### 2.2. Datasets

#### 2.2.1. Microplastic Dataset

The microplastic dataset used in our study originates from the Moore Institute for Plastic Pollution Research and consists of 5653 microscope images. Each image is annotated with labels indicating the types of microplastics present, as well as detailed contours outlining each microplastic within the image.

Microplastics in this dataset are categorized into five types: spheres, fragments, fibers, films, and foams. The definition of these categories, their count and annotations are listed in Table 2.

**Figure 2:**
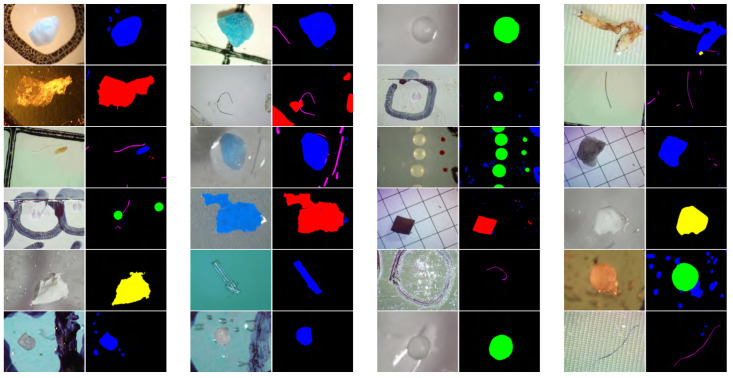
Sample microplastic images and corresponding annotated masks.

**Figure 3:**
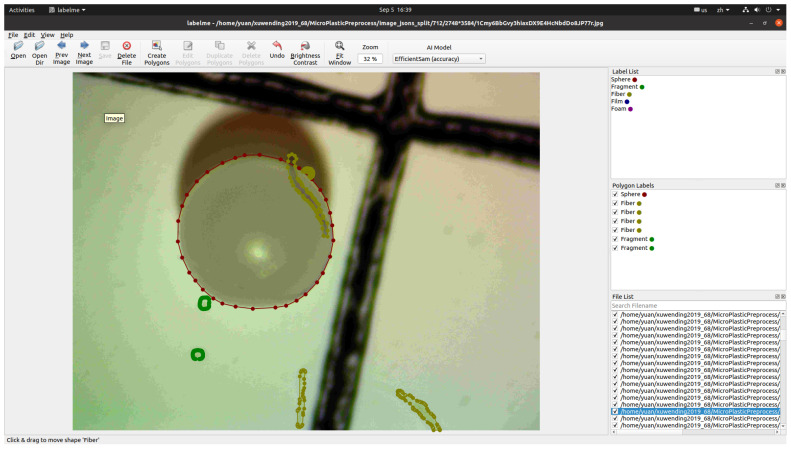
Annotate microplastics in an image using LabelMe.

**Figure 4:**
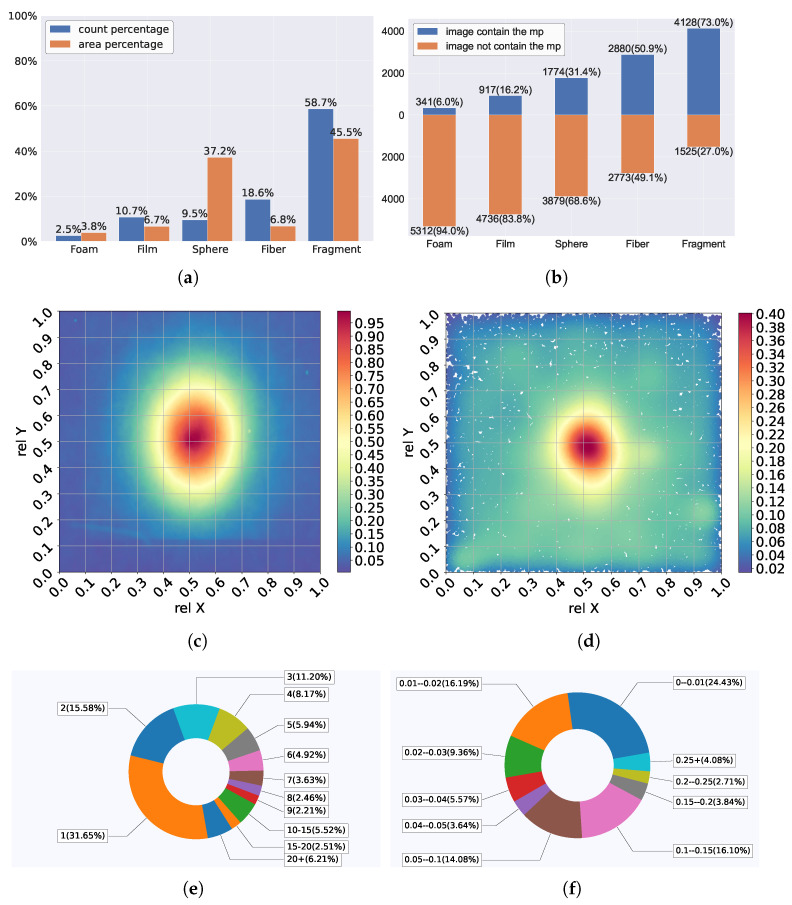
Details of the dataset: (**a**) Percentage of each microplastic’s count and area. (**b**) Percentage of images containing each microplastic. (**c**) Distribution of the mask’s coverage area. (**d**) Distribution of the mask’s center position. (**e**) Breakdown of images by microplastic object count. (**f**) Breakdown of images by microplastic coverage area.

While the dataset provides labels for the type of microplastic in each image, it includes pixel-level segmentation masks. A significant part of this work involves annotating these images to generate precise pixel-level masks, which are essential for training and evaluating semantic segmentation models. Figure 2 shows a list of samples from this dataset as well as their segmentation masks. The annotation tool we use is LabelMe v5.9.0 (https://github.com/labelmeai/labelme, accessed on 23 November 2025), a Python-based tool designed for pixel-level annotation. We utilized its polygon annotation feature to trace the microplastic boundaries, and these polygons were subsequently converted into pixel-level bitmap segmentation masks for model training. A snapshot of the annotation process is shown in Figure 3. After annotation, detailed statistics of the microplastics in this dataset were determined, which are provided in Figure 4.

From Figure 2 and Figure 4, we can see that the dataset encompasses a diverse range of imaging conditions, including variations in size, aspect ratio, magnification, lighting, camera angles, coverage area, center position, background materials, etc.

**Figure 5:**
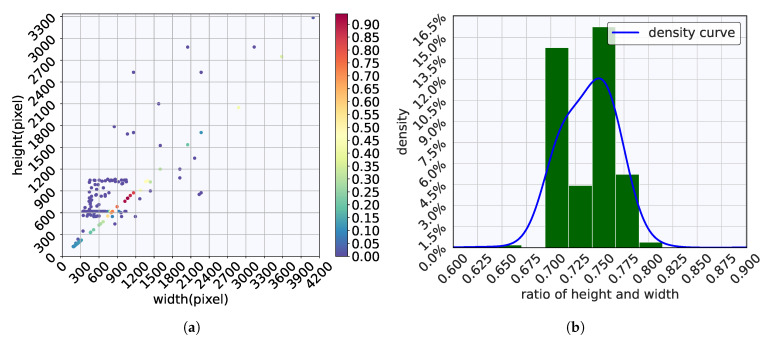
Image size and aspect ratio distributions: (**a**) distribution of image width and height; (**b**) distribution of image aspect ratio.

Figure 5 depicts the distribution of image size and aspect ratio. As shown in Figure 5, the dataset contains images with a wide range of sizes, from as small as 132 × 180 pixels to as large as 3286 × 4096 pixels. The aspect ratios of the images also vary significantly.

This diversity ensures that the dataset is representative of real-world challenges in microplastic detection and analysis, thus enhancing the robustness and generalizability of the models developed.

To facilitate model training and evaluation, the dataset is split into training and validation sets in a 7:3 ratio. Specifically, the training set consists of 3957 images, while the validation set includes 1696 images. This division allows for a comprehensive assessment of the model’s performance on unseen data, providing reliable estimates of its ability to generalize effectively to new and varied samples.

#### 2.2.2. Cityscapes Dataset

As mentioned in Section 2.1.4, we use the Cityscapes dataset to pre-train our model. This is a large-scale dataset designed originally for semantic urban scene understanding. It contains images captured in various street scenes across 50 cities, primarily in Germany. The dataset includes pixel-level annotations for 33 different classes, such as vehicles, pedestrians, buildings, and road markings, making it a comprehensive resource for training models on urban scene segmentation tasks. The images in Cityscapes are captured under diverse conditions, including varying weather, lighting, and traffic scenarios [41], providing a robust foundation for pre-training models that require strong feature extraction capabilities, even if the focus of the target task is different, such as microplastic segmentation. More information on the Cityscapes dataset can be found on the Cityscapes website https://www.cityscapes-dataset.com/, accessed on 23 November 2025.

### 2.3. Data Preprocessing and Augmentation

As shown in Figure 5, the images in the microplastic dataset vary in size, with most being around 400 × 600 or 1200 × 1500 pixels. The majority of these images have an aspect ratio close to 0.75. To standardize the input for our experiments, we resize all images to 640 × 480 pixels. Specifically, we first pad the images to achieve a 4:3 aspect ratio to ensure that objects in the image do not deform significantly, then resize them to 640 × 480. For images smaller than 640 × 480, only the padding step is applied.

In addition to preprocessing, we also apply data augmentation techniques to improve the robustness and generalization of our model. These methods include random rotations, flips, and color jittering, which help the model learn to recognize microplastics under varying conditions and enhance its performance on unseen data. Finally, we generate a series of synthetic images containing microplastics to increase the diversity within the dataset. This approach addresses the imbalance in the original dataset, which contains an overrepresentation of fragment-type microplastics and a limited number of foam- and film-type microplastics, as illustrated in Figure 4.

By diversifying the training data, we aim to reduce overfitting and ensure the model’s effectiveness in the segmentation of microplastics across different imaging scenarios. The augmentation methods used in this work are listed in Table 3.

### 2.4. Evaluation Metrics

In this work, we use a variety of metrics to evaluate our approach. We employ mean Intersection over Union (mIoU) and F1 score (F1) as measures of segmentation accuracy, the value of which ranges from 0 to 1, where a higher value indicates better performance. Besides accuracy metrics, we also employ the number of parameters (params), FLOPs (Floating Point Operations), and inference time to assess the model’s efficiency and computational demands. These metrics provide a detailed understanding of the computational performance of our model. A brief description of these metrics are provided below:

- mIoU: This metric measures the average overlap between the predicted segmentation mask and the ground truth mask. A higher mIoU value indicates better agreement between the predicted and actual segmentation.
- F1: This metric is calculated as the harmonic mean of precision and recall. It provides a balanced measure by considering both false positives and false negatives.
- params: This metric quantifies the number of learnable parameters in a model. While a higher parameter count can improve the model’s capacity to learn complex patterns, it also increases the risk of overfitting and can lead to longer inference times.
- FLOPs: This metric represents the total number of floating-point operations required for a model’s computation. A higher FLOPs value indicates greater computation resource usage.
- Inference time: This metric refers to the average time taken to process a single input and generate an output. A good segmentation model typically achieves an inference time of only a few milliseconds.

## 3. Experiments

### 3.1. Experiment Setup

We trained each model for 300 epochs, with batch size of 8, using the RMSprop optimization algorithm [44]. This batch size was deliberately chosen to ensure a fair comparison across all benchmarked models. Specifically, with an input image resolution of 640×480 pixels, one of the larger comparison models (Nested UNet) reached its maximum stable batch size of 8 on the 24 GB VRAM GPU. Therefore, to maintain identical training conditions and prevent the batch size from influencing comparative results, we standardized the batch size at 8 for all architectures, including our own model.

The learning rate was dynamically adjusted using the Cosine Annealing algorithm, where the learning rate $\eta_{t}$ at epoch *t* is defined as follows:

(4) $$\begin{array}{c} \eta_{t}=\eta_{\min}+\frac{1}{2}\left(\eta_{\max}-\eta_{\min}\right)\left(1+\cos\left(\frac{\pi t}{T_{\max}}\right)\right) \end{array}$$

Here, $\eta_{\min}$ and $\eta_{\max}$ represent the minimum and maximum learning rates, respectively, and $T_{\max}$ denotes the total number of training epochs. In our experiments, we set $\eta_{\min}$, $\eta_{\max}$ and $T_{\max}$ as 0, 0.0001, and 300, respectively. Details of the hardware and software configurations used during training are provided in Table 4.

**Figure 6:**
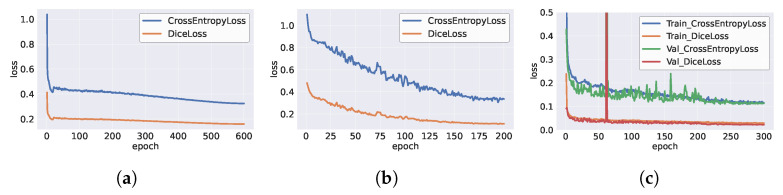
Loss curves on pre-trained and fine-tuned datasets: (**a**) Cityscapes coarse dataset; (**b**) Cityscapes fine dataset; (**c**) MicroPlastics dataset.

As discussed in Section 2.1.4, we pre-trained our model on the Cityscapes dataset before fine-tuning it on the microplastics dataset. The Cityscapes dataset includes 5000 finely annotated images and 19,997 coarsely annotated images, all of which were used in the pre-training phase. The training loss curves for both the Cityscapes and microplastic datasets are illustrated in Figure 6. These curves clearly demonstrate the rapid convergence and low final loss achieved during the pre-training phase and subsequent fine-tuning phase on the microplastic data, validating the effectiveness of our approach.

### 3.2. Comparison Study

To evaluate the performance of our model against other established segmentation approaches, we conducted experiments using a selection of well-known models. Below is a list of these models, along with a brief description of each:

- FCN [45]: The Fully Convolutional Network (FCN) is a pioneering model that introduced the concept of fully convolutional architectures for dense pixel-wise predictions, eliminating the need for fully connected layers.
- BiSeNetV1 [46]: BiSeNetV1 achieves real-time performance by utilizing a dual-path architecture—a spatial path for capturing detailed spatial information and a context path for rich semantic context.
- BiSeNetV2 [47]: This is an enhanced version of BiSeNetV1, featuring a bilateral structure, adaptive aggregation module, and spatial enhancement modules to further improve efficiency and accuracy.
- DeepLabV3+ [38]: Known for its high accuracy, DeepLabV3+ uses an encoder–decoder architecture with atrous convolution and a spatial pyramid pooling module to capture multi-scale contextual information. It offers three backbone options: ResNet, MobileNet, and Xception.
- HRNet [27]: HRNet maintains high-resolution representations throughout the network by employing parallel multi-resolution subnetworks, enabling the capture of both rich spatial details and contextual information.
- U-Net [22]: U-Net utilizes an encoder-decoder architecture with skip connections, which help to preserve spatial information during the segmentation process.
- Nested U-Net [48]: An extension of U-Net, Nested U-Net incorporates a nested and multi-scale architecture to capture hierarchical features, leading to improved segmentation accuracy.
- Swin U-Net [49]: Swin U-Net combines the Swin Transformer with the U-Net architecture, effectively modeling both local and global spatial relationships for enhanced segmentation performance.

This comparison highlights the strengths and innovations of our model relative to these established approaches.

### 3.3. Experiment Results

#### 3.3.1. Visual Comparison

**Figure 7:**
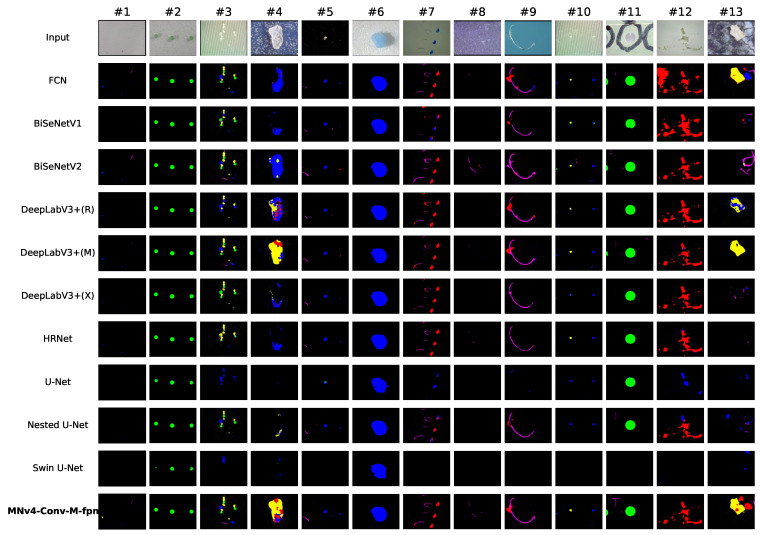
Visual performance comparison of different models.

We present a set of representative visual segmentation results for all models in Figure 7. The figure shows that for spherical and fragment-shaped microplastics, such as in inputs 2, 6, and 11, most models perform well, accurately segmenting these objects due to their distinct shapes, consistent textures, and simple background interactions.

However, film and fiber-shaped microplastics, like those in inputs 9 and 12, pose significant challenges for most models. Fiber-shaped microplastics are typically thin, leading to discontinuous segmentation results, while film-shaped microplastics are often transparent, making them difficult to distinguish from the background. The majority of models also struggle to segment foam-shaped microplastics, as seen in inputs 4 and 13. This is somewhat counter-intuitive, given that foam microplastics are visually identifiable, but their underrepresentation in the dataset—only 6% of the images contain at least one foam-shaped microplastic—likely contributes to this difficulty. Despite this, our model is able to produce accurate segmentations for inputs 4 and 13, demonstrating its strong capability to handle images with underrepresented training samples.

As shown in Figure 7, our model excels at detecting and accurately delineating the boundaries of small-sized microplastics, such as in inputs 9 and 10, where several other models fall short. This superior performance is attributed to the incorporation of a feature pyramid network and a feature fusion module in our model, which enable it to effectively capture small objects and integrate information across different resolutions for enhanced boundary detection.

#### 3.3.2. Evaluation Results

**Figure 8:**
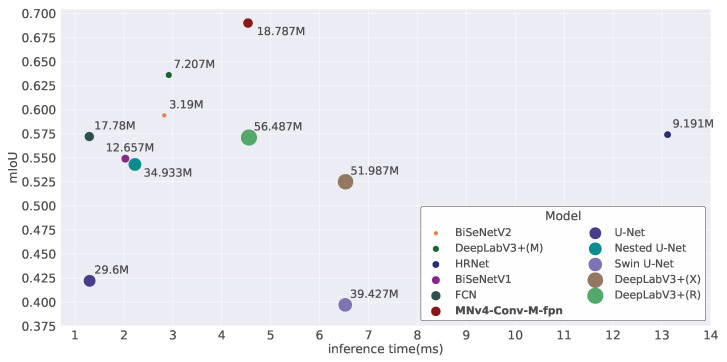
mIoU, params and inference time for each model.

To accurately compare the performance of our model with the models listed in Section 3.2, we present detailed results on the evaluation metrics described in Section 2.4 in Table 5 and Figure 8.

As shown in Table 5, our model outperforms all others in both mIoU and F1 score, indicating it is the most accurate among those tested. Additionally, our model has the lowest FLOPs, demonstrating its efficiency in terms of computational power. In contrast, while Swin U-Net has more than twice the number of parameters as our model, it performs poorly in both accuracy and inference time, suggesting that the Swin Transformer may not be the best choice for segmenting microplastics.

This is further illustrated in Figure 8, where our model surpasses the second-best model by as much as 8.5% in mIoU while maintaining a 4.5 ms inference time, making it fast enough for real-time applications.

To further demonstrate our model’s effectiveness in segmenting various types of microplastics, we present detailed mIoU and F1 scores for each type of microplastic across all models in Table 6. From this table, it is evident that our model outperforms all others in accuracy across all microplastic types. Notably, for fiber-shaped microplastics, it is the only model to achieve an F1 score above 0.75.

### 3.4. Ablation Study

To evaluate the contribution of each component in our model, we conducted an ablation study by systematically removing specific modules and observing the impact on performance. This analysis helps identify which modules are critical to the model’s success in segmenting microplastics images, providing deeper insights into its design and effectiveness.

Our model comprises three main components: a backbone network, a feature pyramid network, and a feature fusion module. We used the backbone network (MNv4-Conv-M) as the baseline and focused this ablation study on the effects of the feature pyramid network and the feature fusion module.

**Figure 9:**
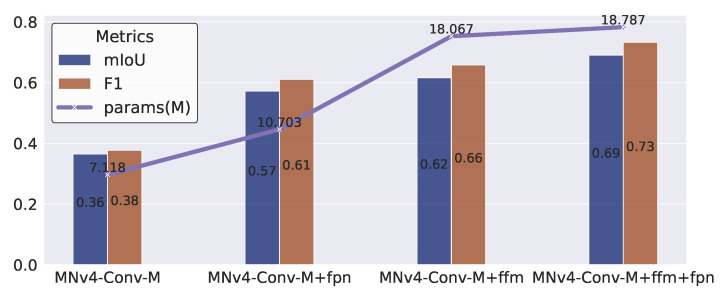
Ablation experiment.

The results of this experiment are presented in Figure 9. It is evident that integrating the feature pyramid network into the baseline model leads to a substantial increase in mIoU, from 0.36 to 0.57, and a corresponding rise in F1 score, from 0.38 to 0.61. The subsequent addition of the feature fusion module further boosts the mIoU and F1 score to 0.69 and 0.73, respectively. These findings clearly demonstrate that both the feature pyramid network and the feature fusion module play crucial roles in significantly enhancing the accuracy of our model. Moreover, the synergy between the feature pyramid network and the feature fusion module further amplifies their individual contributions to performance improvement.

To assess the impact of transfer learning, we compared the performance of the full MNv4-Conv-M-fpn model when trained from scratch versus when trained using weights pre-trained on the Cityscapes dataset. Training the model from scratch yields an mIoU of 0.655 and an F1 score of 0.696. In contrast, incorporating the Cityscapes pre-training step results in the highest achieved performance: an mIoU of 0.690 and an F1 score of 0.733. This performance lift confirms that pre-training is a highly effective and necessary component of our methodology.

## 4. Discussion

### 4.1. The Scientific and Technical Advancement

This work introduces MNv4−Conv−M−fpn, a novel deep learning architecture that significantly advances the capability for automated microplastic analysis. To the best of our knowledge, our study is the first to integrate advanced deep learning components, specifically MobileNetV4, Feature Pyramid Networks, and a Feature Fusion Module, for multi-category microplastic segmentation under varied and complicated backgrounds. This approach directly addresses a major analytical bottleneck in marine science.

Most existing studies on automated microplastic identification focus on binary segmentation, distinguishing only between microplastic and background. In stark contrast, our methodology focuses on the far more complex task of segmenting images into six categories, including five distinct microplastic morphologies and the background. This granular, multi-class segmentation poses a significantly greater technical challenge but is essential for robust marine pollution measurement and analysis. Morphological differentiation allows researchers to link pollution findings to specific sources (e.g., distinguishing fibers from fragments or pellets), which is critical for effective source control and management.

Furthermore, much of the related research relies on U-Net or its variants for segmentation. Our approach introduces a more advanced deep learning model, which, as demonstrated in Section 3.3.2, consistently outperforms U-Net in accuracy. To ensure a comprehensive assessment against contemporary AI tools, we compared MNv4−Conv−M−fpn with other state-of-the-art deep convolutional neural network models using established metrics such as mean Intersection over Union (mIoU), F1 score, number of parameters, FLOPs, and inference time. These rigorous comparisons confirm that our model is not only highly accurate but is also lightweight and capable of real-time processing, a crucial feature for field deployability. An ablation study was also conducted to rigorously verify the unique contribution of each architectural component to the overall performance improvement, proving that the synergistic design of our proposed model is the most suitable for high-performance microplastic segmentation.

While deep learning has also been applied to microplastic classification—for example, through standardized visual protocols [50], holographic methods [51], and models like SqueezeNet or ResNet [52]—segmentation provides superior environmental insight. Classification provides only overall category information without specifying the location or size of particles. In contrast, segmentation effectively differentiates microplastics from complex backgrounds, accurately identifying the type, precise location, shape, and area of each particle. While technically more challenging, segmentation offers more detailed and useful insights essential for accurate ecotoxicological and risk assessment and tracking the fate of pollutants in marine environments.

### 4.2. Environmental Deployment and Policy Impact

The high computational efficiency and real-time processing capabilities of MNv4-Conv-M-fpn significantly enhance its utility for large-scale exposure assessment and ecotoxicological monitoring. This lightweight architecture is specifically designed for deployment on mobile and embedded platforms, where portability and efficiency are paramount for scalable research. By integrating this advanced AI tool into field devices, researchers and environmental agencies can perform rapid, on-site microplastic analysis. This capability circumvents the reliance on extensive and time-consuming laboratory infrastructure, which has historically created a bottleneck in collecting the high-volume data needed for robust risk modeling and understanding the full extent of microplastic hazard. This streamlined process enables more immediate and widespread characterization of microplastic pollution on a global scale.

This high-efficiency approach offers a direct pathway to improve the risk management and protection of the natural environment. Generating real-time, morphology-specific data supports more agile policy decisions, allowing for the rapid identification of microplastic hotspots and the efficient targeting of mitigation efforts. Crucially, the ability to characterize microplastics by shape and size in real time provides data essential for understanding differential exposure routes and predicting toxicological consequences based on particle morphology. Furthermore, the adaptability of this segmentation approach provides a foundation for broader applications, such as the real-time detection of other morphologically distinct marine pollutants or contaminants, further extending its value as a novel tool for environmental toxicology.

### 4.3. Limitations and Future Work

Despite the strong capabilities demonstrated, our model has certain limitations that warrant further attention. The segmentation performance on some microplastic types is constrained by dataset imbalance, particularly for underrepresented categories such as foam. To address this, future work will focus on the collection of additional high-quality microscope images containing a broader diversity of challenging microplastic morphologies. Furthermore, the segmentation of transparent objects, such as film microplastics, is currently less effective due to background interference. We plan to optimize the model’s architecture and loss function to specifically improve the accurate segmentation of transparent objects. Additionally, we aim to further enhance the model’s performance and efficiency by incorporating the latest advancements in computer vision, such as SAM2 [53], ensuring the model remains at the forefront of automated marine environmental analysis.

## 5. Conclusions

In this work, we introduced MNv4-Conv-M-fpn, a novel deep learning model engineered for accurate and morphologically-specific multi-class microplastic segmentation, directly addressing the measurement bottleneck in environmental analysis. By combining the lightweight MNv4-Conv-M backbone with a Feature Pyramid Network and a dedicated Feature Fusion Module, our model achieves high accuracy in multi-class microplastic detection, while maintaining a low computational load and fast inference speed. This efficiency makes the model ideal for high-throughput deployment in environmental laboratories and resource-constrained monitoring efforts, providing the granular morphological data essential for differential toxicological studies and risk assessment. This innovative approach serves as a valuable, scalable tool for ecotoxicological monitoring, exposure assessment, and informed environmental risk management.

## Figures and Tables

**Table 1:** Summary of deep learning works in microplastic image segmentation, detailing the scope of particle measurement and analysis.

Research Work	Number of Categories	Model
Ji Yeon Baek et al. [21]	2	U-Net
Gwanghee Lee et al. [23]	2	U-Net, MultiResU-Net
Ka Shing Lee et al. [30]	2	U-Net
Ho-min Park et al. [31]	2	U-Net
Bin Shi et al. [32]	2	MultiResU-Net
Hui Huang et al. [17]	2	ResNet + FPN
Jiongji Xu et al. [26]	2	U-Net, U-Net2Plus, U-Net3Plus
Sarah-Jeanne Royer et al. [29]	5	ResNet
Jaeheon Jeong et al. [28]	2	HRNet
**Our Work**	**6**	**MNv4+FPN+FFM**

**Table 2:** Detailed information of each MP category.

Category	Description	Count	Color
Sphere	Round, smooth particles; clear or colored	3220	green
Fragment	Irregular, jagged pieces; dull	19,871	blue
Fiber	Thread-like, colorful structures	6312	magenta
Film	Thin, flexible, transparent sheets	3626	red
Foam	Light, spongy texture; usually white	851	yellow

**Table 3:** Image augmentation methods.

Method	Description
Rotation	Rotate the input image by angle with a given probability.
ColorJitter	Change the brightness, contrast, saturation and hue of an image.
GaussianBlur	Blurs image with a given Gaussian blur kernel.
VerticalFlip	Flip the input image vertically with a given probability.
HorizontalFlip	Flip the input image horizontally with a given probability.
ElasticTransform	Randomly offset pixels with given α and σ.

**Table 4:** Configuration of the experiment.

Component	Configuration
CPU	Intel(R) Core(TM) i9-14900K
GPU	GeForce RTX4090 (24 GB)
Memory	64 G
Hard Disk	2 T
Operating System	Ubuntu 20.04.6 LTS
CUDA	12.4
Driver	NVIDIA-550.90.07
Pytorch	2.2.0

**Table 5:** Model’s overall performance on metrics.

Model	mIoU	F1	Inference Time (ms)	Params (M)	Flops (B)
FCN	0.572	0.612	**1.292**	17.780	11.957
BiSeNetV1	0.549	0.578	2.029	12.657	1.744
BiSeNetV2	0.594	0.633	2.825	**3.190**	1.449
DeepLabV3+(R)	0.571	0.604	4.556	56.487	10.378
DeepLabV3+(M)	0.636	0.677	2.917	7.207	3.968
DeepLabV3+(X)	0.525	0.556	6.531	51.987	6.329
HRNet	0.574	0.609	13.115	9.191	2.189
U-Net	0.422	0.445	1.298	29.600	25.668
Nested U-Net	0.543	0.580	2.225	34.933	65.007
Swin U-Net	0.397	0.416	6.525	39.427	3.487
**MNv4-Conv-M-fpn**	**0.690**	**0.733**	4.537	18.787	**1.283**

**Table 6:** mIoU and F1 score breakdown for each model across different microplastic types.

Model	Fiber	Film	Foam	Fragment	Sphere
FCN	0.640∣0.698	0.625∣0.685	0.657∣0.731	0.700∣0.741	0.720∣0.775
BiSeNetV1	0.596∣0.647	0.640∣0.713	0.659∣0.739	0.644∣0.680	0.705∣0.764
BiSeNetV2	0.635∣0.695	0.643∣0.713	0.663∣0.741	0.672∣0.713	0.715∣0.777
DeepLabV3+(R)	0.610∣0.664	0.639∣0.710	0.661∣0.740	0.665∣0.703	0.707∣0.766
DeepLabV3+(M)	0.647∣0.710	0.647∣0.717	0.664∣0.742	0.706∣0.747	0.731∣0.791
DeepLabV3+(X)	0.598∣0.648	0.635∣0.705	0.660∣0.740	0.645∣0.682	0.678∣0.743
HRNet	0.614∣0.669	0.642∣0.714	0.663∣0.742	0.667∣0.706	0.710∣0.768
U-Net	0.581∣0.623	0.638∣0.710	0.655∣0.734	0.582∣0.611	0.643∣0.691
Nested U-Net	0.622∣0.678	0.629∣0.695	0.651∣0.727	0.670∣0.709	0.711∣0.768
Swin U-Net	0.580∣0.623	0.638∣0.710	0.655∣0.734	0.554∣0.585	0.622∣0.682
**MNv4-Conv-M-fpn**	**0.684∣0.750**	**0.649∣0.719**	**0.667∣0.744**	**0.733∣0.777**	**0.741∣0.802**

## Data Availability

The raw data supporting the conclusions of this article will be made available by the authors on request.
